# Supplementary figures and images for: Modeling the Dynamics of Bivalent Histone Modifications
Source: PLoS One. 2013 Nov 1;8(11):e77944. doi: 10.1371/journal.pone.0077944 (PMC3815350; doi:10.1371/journal.pone.0077944)

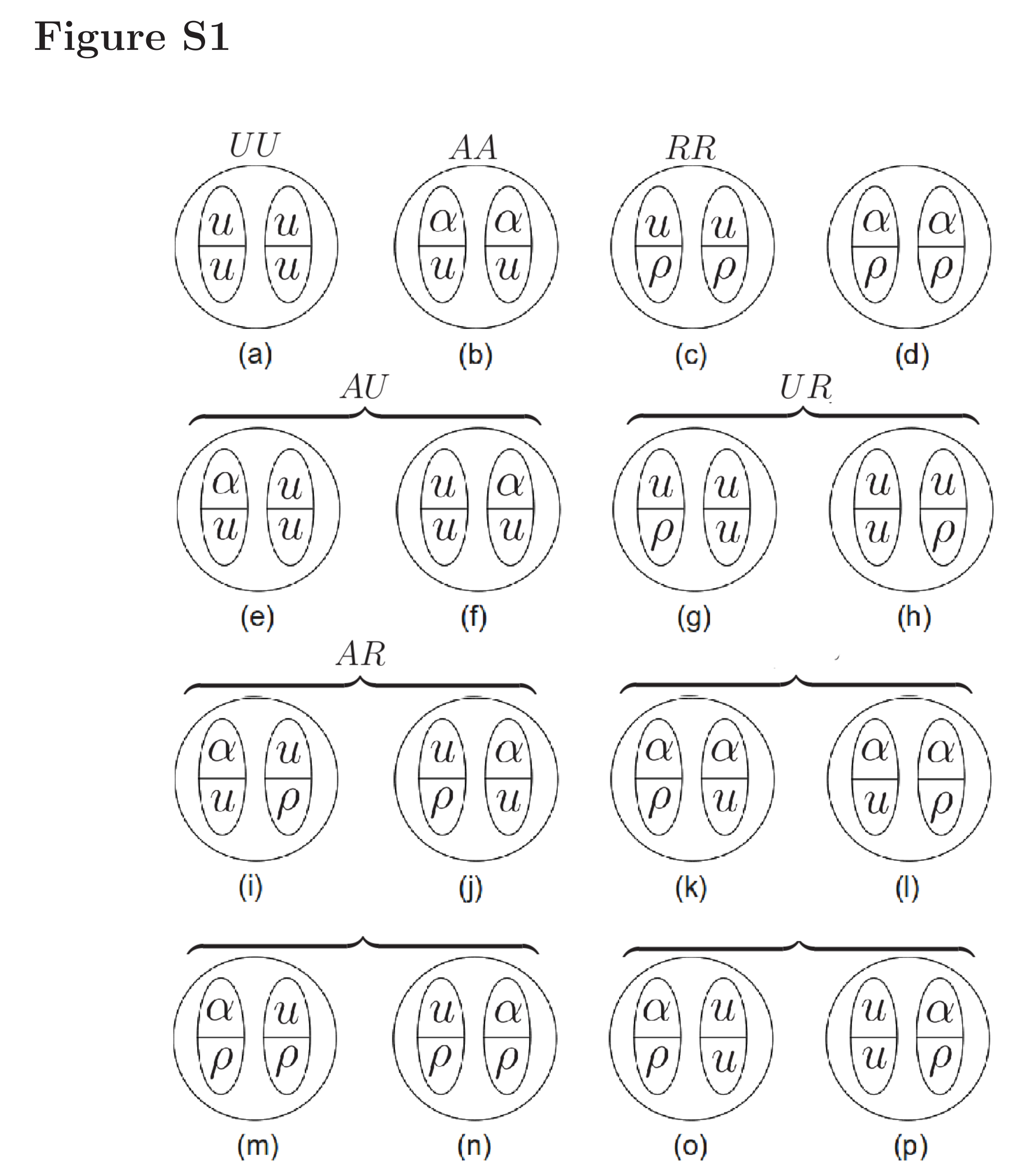

Supplement: Figure S1 — Illustration for the explanation of the states of the 6-state model. (TIFF) [file pone.0077944.s001.tiff]

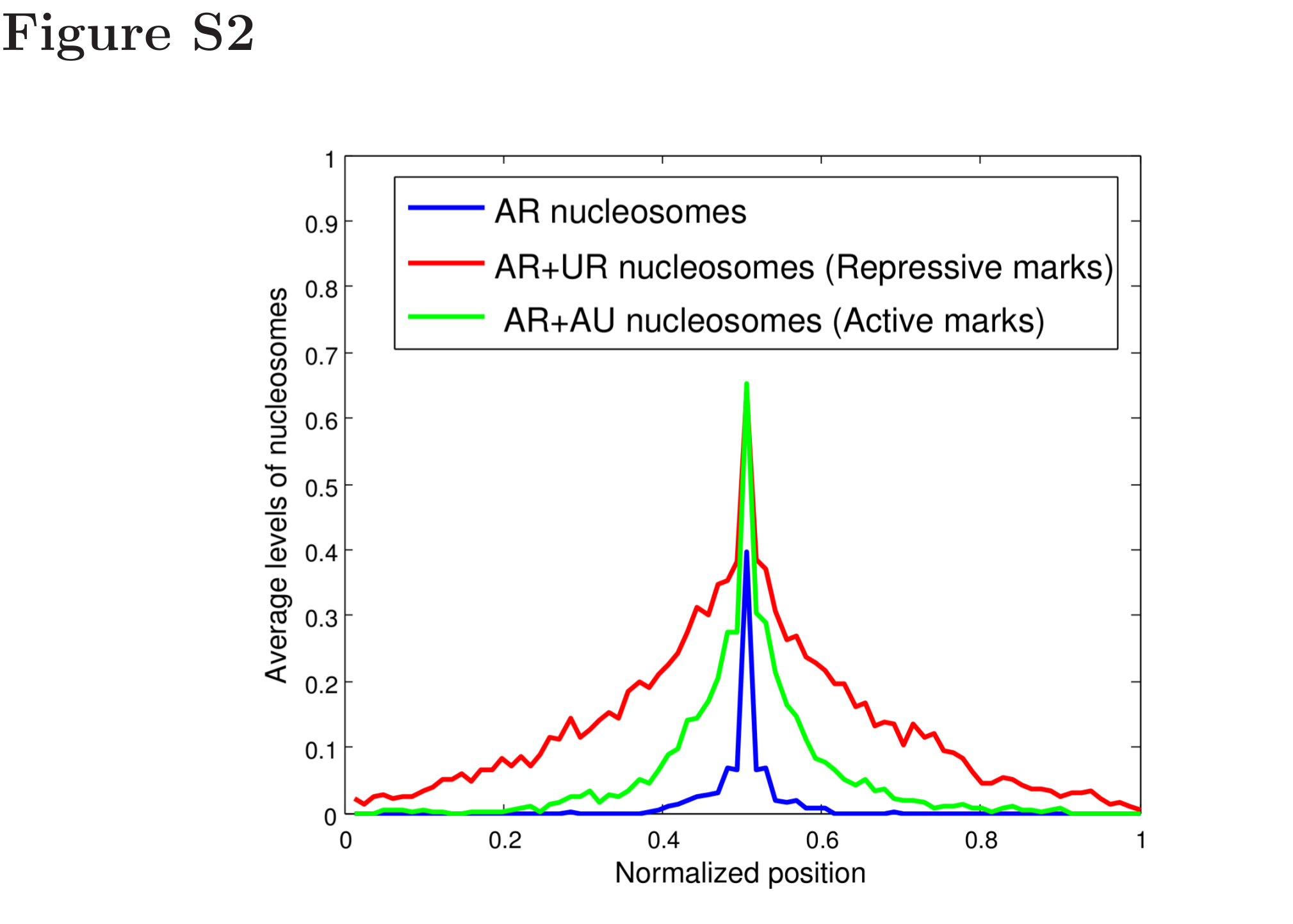

Supplement: Figure S2 — An example of the distribution of nucleosomes, active, and repressive marks. This plot illustrates that the 4-state model described in the main text can simulate bivalent domains (blue) in which the active mark (green) is less extensive than the repressive mark (red) (i.e., the bivalent domains (blue) are buried in the repressive domains (red)). The details of simulation can be referred to Section 4.3 in the main text. Here, distributions of nucleosomes (blue), nucleosomes (red), and nucleosomes (green) are plotted at the end of the simulation runs (time = 1800). The average levels of nucleosomes are averaged over 1000 simulation runs. In the simulation, and . The other parameters are , , , , and . (TIFF) [file pone.0077944.s002.tiff]
