# Supplementary material for: Modeling the Dynamics of Bivalent Histone Modifications
Source: PLoS One. 2013 Nov 1;8(11):e77944. doi: 10.1371/journal.pone.0077944 (PMC3815350; doi:10.1371/journal.pone.0077944)
Supplement: Text S1 — The supplementary text provides details regarding the formulation of our model and another example of localization of states related to our results in Section 4.3. (PDF) [file pone.0077944.s003.pdf]

Supplementary Text S1

Model

6-state model. Refer to Fig. S1. Circles in the figure represent nucleosomes. A nucleosome contains two histone copies represented by the vertically oriented ellipses. Each histone has a site (represented by the upper half of the ellipse) that can be either unmodified (symbolized by u) or have an active mark (symbolized by α) and another site (represented by the lower half of the ellipse) that can be either unmodified (symbolized by u) or have a repressive mark (symbolized by ρ). Each of the four modification sites in a nucleosome can be in one of two states (modified or unmodified), yielding the $2^4 = 16$ possibilities that are shown in the figure panels, (a)-(p). Panels grouped together by the curly brackets in the figure represent the same physical nucleosome state, e.g., panels (e) and (f) are considered to represent the same physical nucleosome state since (f) results from (e) by interchange of the left and right histone ellipses. There are six such pairs. Thus there are 10 physically distinct nucleosome states. In addition, experiments indicate that active and repressive marks do not occur simultaneously on the same histone [1] (i.e., α and ρ do not occur in the same ellipse). This eliminates the possibilities depicted in panels (d) and (k)-(p). Thus we arrive at 6 possible states. In these 6 possible states, each histone has three distinct configurations, and we assign symbols A, U, R to them. They have the following meanings.

A**:** The histone has an active mark and the other site is unmodified.

U**:** All two sites are unmodified.

R**:** The histone has a repressive mark and the other site is unmodified.

As a result, the six possible states can be depicted by 2 letters instead of 4 letters, which we label UU, AA, RR, AU, UR, and AR as shown in Fig. S1.

Reduced model. We now introduce a reduction of the above 6-state model to a more simple model. Our reduction is motivated by a limited number of simulations of the 6-state model in which we found that the experimentally observed bivalent nucleosome state (AR) tended to be absent unless the AA and/or RR states were suppressed (i.e., $\pi_{\sigma\sigma'}$ is low for the transition $\sigma = AU \to \sigma' = AA$ and the transition $\sigma = UR \to \sigma' = RR$). This is consistent with a recent experimentally motivated hypothesis that the existence of the asymmetrically modified nucleosome states, AU and UR, are important for the formation of bivalent domains [1].

One way of understanding this is to note from Fig. 2 that the AA state competes with the AR state for conversion from the AU state, and the RR state similarly competes with the AR state for conversion from the UR state. This suggests that if we want to allow for

the occurrence of the experimentally observed AR state, we could chose parameters in our six state model such that the transition rate from AU to AA is sufficiently smaller than the transition rate to AR. Similarly we would want the transition rate from UR to RR to be sufficiently smaller than the transition rate to AR. Thus, to make the model more tractable, we employ a further simplification and consider the idealized case in which AA and RR are completely suppressed. That is, in terms of our 6-state model, we set $\pi_{\sigma\sigma'} = 0$ for the transition $\sigma = AU \rightarrow \sigma^{'} = AA$ and the transition $\sigma = UR \rightarrow \sigma^{'} = RR$. In this formulation, AA and RR states do not occur, and the 6-state model reduces to a 4-state model.

Another example of localization of AR states related to our results in Section 4.3

We note that our result in Fig. 9 is not consistent with experiment in that in Fig. 9 the active marks are more extensive than the repressive marks, while Ref. [2] shows that the opposite situation holds in experiment. We note, however, that, as shown in Fig. S2, for other reasonable parameter choices, we can also obtain states for which the repressive marks are more extensive than the active marks (consistent with [2]).

References

1. Voigt P, LeRoy G, III WD, Zee B, Son J, et al. (2012) Asymmetrically modified nucleosomes. Cell 151: 181 - 193.

2. Bernstein BE, Mikkelsen TS, Xie X, Kamal M, Huebert DJ, et al. (2006) A Bivalent Chromatin Structure Marks Key Developmental Genes in Embryonic Stem Cells. Cell 125: 315.
